# Non-alcoholic fatty liver disease frequency and associated factors at admission of acute stroke

**DOI:** 10.1007/s12072-021-10253-z

**Published:** 2021-09-15

**Authors:** Takahisa Mori, Kazuhiro Yoshioka, Yuhei Tanno

**Affiliations:** Department of Stroke Treatment, Shonan Kamakura General Hospital, Okamoto 1370-1, Kamakura City, Kanagawa 247-8533 Japan

**Keywords:** Albumin, AST/ALT ratio, Blood pressure, BMI, DGLA, HbA1c, Hemorrhagic stroke, Ischemic stroke, Palmitic acid, Predictor

## Abstract

**Background/purpose of the study:**

If non-alcoholic fatty liver disease (NAFLD) frequency is very high in stroke patients, NAFLD may be a risk factor for stroke and identifying factors of NAFLD presence may lead to stroke prevention. This retrospective study aimed to investigate whether NAFLD frequency was very high and identify factors associated with NAFLD presence at acute stroke admission.

**Methods:**

We included stroke patients aged 40 − 79 years who (1) were admitted from 2016 to 2019, within 24 h of onset; (2) underwent abdominal ultrasonography; and (3) underwent blood examination of biomarkers. We evaluated the frequency and significant factors of NAFLD presence.

**Results:**

Among 1672 stroke patients, 676 patients met our inclusion criteria, and 267 patients (39.5%) had NAFLD. Compared to patients without NAFLD, patients with NAFLD were young; had high anthropometric values; high blood pressure; low aspartate aminotransferase/alanine aminotransferase ratio (AST/ALT) ratio; high levels of liver enzymes, serum albumin, HbA1c, and serum lipids; low-density lipoprotein; high serum level of some fatty acids; and high fatty acid% of palmitic acid (PA) and dihomo-gamma-linolenic acid (DGLA). After excluding variables with multicollinearity, independent NAFLD-presence factors were high body mass index (BMI), low AST/ALT ratio, high serum albumin level, high PA%, and high DGLA level.

**Conclusions:**

The frequency of NAFLD was high in our patient group. Significant NAFLD-presence factors were high BMI, low AST/ALT ratio, high serum albumin level, high PA%, and high DGLA level. A further study is warranted to determine the effects of the NAFLD-presence factors on stroke onset or prevention.

**Supplementary Information:**

The online version contains supplementary material available at 10.1007/s12072-021-10253-z.

## Introduction

Non-alcoholic fatty liver disease (NAFLD) is emerging as one of the most common chronic liver diseases. Metabolic syndrome is a strong predictor of NAFLD that frequently occurs with obesity, type 2 diabetes mellitus, dyslipidemia, and hypertension [1, 2]. High body mass index (BMI), high triglyceride level, low high-density lipoprotein cholesterol (HDL) level, and high glycated hemoglobin (HbA1c) level strongly suggest the presence of NAFLD [3]. NAFLD has been reported to be a predictor of cardiovascular disease and stroke [2, 4]. Liver enzymes, such as aspartate aminotransferase (AST), alanine aminotransferase (ALT), and gamma-glutamyltransferase (GGT) have been associated with the development of cardiovascular disease and all-cause mortality [5]. AST/ALT ratio is a significant predictor of chronic heart failure with reduced left ventricular ejection fraction and cardiovascular mortality [6, 7].

NAFLD may predispose one to ischemic and hemorrhagic stroke [8, 9]. A cross-sectional study conducted from 2011 to 2012 in the general Japanese population demonstrated that the prevalence of NAFLD defined by ultrasonography (US) was 26.4% [10]. Previous retrospective studies reported that the prevalence of NAFLD defined by increased AST or ALT was 7.7% or 42.5%, respectively, in patients with ischemic stroke [11, 12]; however, the prevalence of NAFLD diagnosed by US in patients with acute stroke is unknown. Adults aged 40–79 years have higher risks of metabolic syndrome and cardiovascular disease than individuals of other ages [13], whereas the etiology in stroke patients aged < 40 or ≥ 80 years is frequently different from that in patients aged 40–79 years, e.g., autoimmune disease, atrial fibrillation, or cancer-related thrombosis [14, 15]. Stroke should be aggressively prevented in adults aged 40–79 years with metabolic syndrome or NAFLD. Dietary triglycerides (TG), which are derived from meat, fish, and vegetable oil, influence serum TG and fatty acids (FA) levels. TG, a predictor of NAFLD [10], comprises glycerol and three FAs; thus, the quality of TG depends on FAs. Elevated serum palmitic acid (PA) and oleic acid levels are associated with an increased frequency of lacunar stroke, while elevated serum docosahexaenoic acid (DHA) and arachidonic acid (AA) levels are associated with a decreased incidence of ischemic stroke [16]. Elevated serum dihomo-gamma-linolenic acid (DGLA) level and decreased DHA weight percentage (%) of total fatty acids are associated with the onset of acute ischemic stroke at a younger age [17]. In addition, increased DGLA level and decreased eicosapentaenoic acid (EPA) percentage are associated with the onset of intracerebral hemorrhage (ICH) in young patients [18]. Dyslipidemia is related to NAFLD, and serum FAs are associated with the age of stroke onset [1, 2, 17, 18]; therefore, serum FAs may be associated with NAFLD in patients with stroke. In addition, BMI, triglycerides, type 2 diabetes mellitus, AST/ALT ratio, GGT, and blood pressure may be associated with NAFLD in patients with stroke. If the frequency of NAFLD diagnosed by US is very high in patients with acute ischemic or hemorrhagic stroke, NAFLD may be a critical risk factor for acute stroke. Therefore, identifying the factors associated with NAFLD in patients with acute stroke may lead to stroke prevention. The purpose of this retrospective cross-sectional study was to investigate whether the frequency of NAFLD diagnosed by US was high and identify the factors associated with NAFLD in patients aged 40–79 years with acute stroke.

## Materials and methods

We included patients with stroke who were admitted to our institution from May 2016 to July 2019. We excluded patients who (1) were younger than 40 years or older than 79 years; (2) had an onset-to-door time of > 24 h; (3) did not undergo body weight measurement; (4) did not undergo abdominal ultrasonography within 5 days of onset of symptom; (5) did not undergo blood examination for serum fatty acids at admission; or (6) had alcoholic fatty liver disease.

### Evaluation

We evaluated age; sex; ischemic or hemorrhagic stroke; anthropometric variables, such as body weight, body height, and BMI; variables of blood pressure such as systolic blood pressure (SBP), diastolic blood pressure (DBP), and mean blood pressure; biomarkers, such as serum glucose, HbA1c, total cholesterol, low-density lipoprotein (LDL), HDL, triglycerides, AST, ALT, AST/ALT ratio, and GGT; serum concentration of FAs such as saturated fatty acids, lauric acid, myristic acid, PA, and stearic acid; *n*-9 monounsaturated fatty acids such as oleic acid; *n*-6 polyunsaturated fatty acids (PUFAs), such as linoleic acid (LiA), DGLA, and arachidonic acid (AA); *n*-3 PUFAs such as alpha-linolenic acid (AlA), eicosapentaenoic acid, and docosahexaenoic acid; and FA%. LDL concentration was calculated using the Friedewald formula: LDL = total cholesterol − HDL – triglycerides/5. Mean blood pressure was calculated with the following formula: mean blood pressure = (SBP − DBP)/3 + DBP.

### Measurement of fatty acids

The levels of FAs in 1 mL of serum were measured at BML, Inc. (Shibuya, Tokyo, Japan). FAs were extracted using tricosanoic acid (Nu-Chek Prep, Inc., Elysian, MN, USA) as an internal standard. Lipid extracts were hydrolyzed, extracted with chloroform, and dried under nitrogen gas. After 30% potassium methoxide methanol solution (FUJIFILM Wako Pure Chemical Corporation, Osaka, Osaka, Japan) was added to the residual sample, it was incubated at 100 °C for 5 min, then cooled. Samples were extracted with hexane and analyzed on a GC-2010 Plus Capillary Gas Chromatograph (SHIMADZU Corporation, Kyoto, Japan) equipped with a flame ionization detector and a BPX70 column (30 m × 0.22 mm I.D., 0.25-μm film thickness; SHIMADZU GLC Ltd., Tokyo, Japan). Component identification was performed by comparing retention times with those of the respective standards (Sigma-Aldrich Japan, Inc., Meguro, Tokyo, Japan; Nu-Chek Prep, Inc., Elysian, MN, USA). The serum concentrations of the FAs were determined using internal standard ratios.

### Diagnosis of fatty liver and criterion for diagnosing “non-alcoholic” disease

Fatty liver was diagnosed according to the findings of abdominal conventional B-mode ultrasonography performed by trained technicians. Of four known criteria (hepatorenal echo contrast, liver brightness, deep attenuation, and vascular blurring), hepatorenal echo contrast and liver brightness were used to diagnose fatty liver [19, 20]. In addition, when the daily alcohol (“ethanol”) consumption was lower than 30 g in men and 20 g in women, a positive NAFLD diagnosis was made [20].

### Statistical analysis

The chi-square test was used to compare the categorical variables. We expressed non-normally distributed continuous variables as medians and interquartile ranges. The Wilcoxon rank-sum test was used to compare continuous variables in unpaired groups by normal approximation. We compared all possible pairs of variables with significant differences between patients with NAFLD (NAFLD presence group) and patients without NAFLD (NAFLD absence group). A dummy variable was used to represent categorical data, such as data on sex, and Spearman’s rank correlation coefficient (*r*_s_) was calculated to measure the strength of the relationships. We defined 0 ≤|*r*_s_|< 0.1 as no correlation, 0.1 ≤|*r*_s_|< 0.5 as weak correlation, and 0.5 ≤|*r*_s_| as strong correlation. Variable candidates for multiple logistic regression analyses were those with significant differences in the Wilcoxon rank-sum test between the two groups. We adopted the variable candidate with a smaller or the smallest probability (*p*) value or a larger or the largest absolute value of the Wilcoxon rank-sum test statistic (|*z*|) between or among variable candidates with mutually strong correlations. Multicollinearity was defined as variance inflation factor (VIF) ≥ 3. Using variables without multicollinearity, we conducted multiple logistic regression analyses to identify independent variables that distinguish NAFLD presence from NAFLD absence. We estimated the threshold values of independent variables for NAFLD presence using the area under the curve values derived from receiver operating characteristic curves of the logistic regression model. A *p* value < 0.05 was considered statistically significant. We used the JMP software (version 16.0; SAS Institute, Cary, NC, USA) for all statistical analyses. One author (TM) had full access to all the data in the study and took responsibility for its integrity and the data analysis.

## Results

**Fig. 1:**
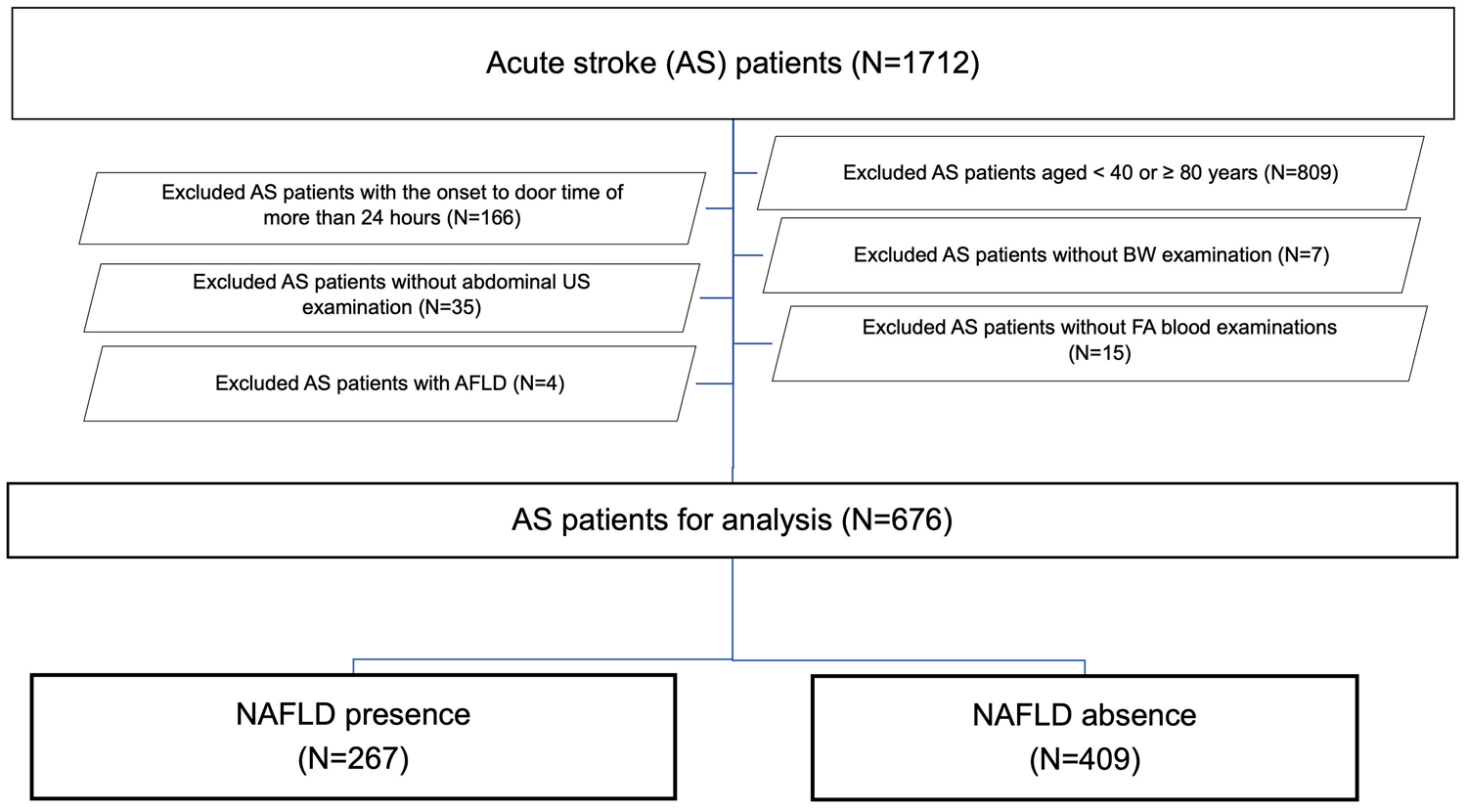
Flow chart of patient selection for the analysis. *AFLD* alcoholic fatty liver disease, *BW* body weight, *FA* fatty acid, *NAFLD* non-alcoholic fatty liver disease; US, ultrasonography

Among 1712 patients with stroke admitted to our institution during the study period, 676 patients met our inclusion criteria for analysis (Fig. 1). Patient characteristics are summarized in Supplemental Tables 1 and 2. NAFLD was diagnosed in 267 patients (39.5%). Compared to patients in the NAFLD absence group, patients in the NAFLD presence group were young, had high anthropometric values, had high levels of liver enzymes, had a low AST/ALT ratio, had high levels of serum albumin and HbA1c, had high levels of serum lipids, excluding LDL, and had high BP (Table 1). Further, patients in the NAFLD presence group had high levels of serum fatty acids, except for EPA; high fatty acid% of lauric acid, myristic acid, PA, oleic acid, and DGLA; and low fatty acid% of stearic acid, linoleic acid, and arachidonic acid (Tables 1 and 2). There were no differences in proportions of ischemic stroke or history of statin use between the two groups (Table 1). After evaluating variables with mutually strong correlations among 36 variables with significant differences between the 2 groups (Tables 1 and 2, Supplemental Tables 3 to 12), 17 variables were adopted as variable candidates for multiple logistic regression, and VIF < 3 in the 17 variable candidates was confirmed (Supplemental Table 13). Multiple logistic regression analysis using the 17 variables showed that BMI, AST/ALT ratio, albumin, PA%, and DGLA were independent NAFLD-presence variables (Table 3). Receiver operating characteristic curves demonstrated that the threshold values of BMI, AST/ALT ratio, albumin, PA%, and DGLA for NAFLD presence were ≥ 23.7 kg/m^2^, ≤ 1.25, ≥ 41 g/L, ≥ 24.5%, and ≥ 97.1 μmol/L, respectively (Table 4). These parameters (BMI, AST/ALT ratio, albumin, PA%, and DGLA) might be NAFLD-presence risk factors at their threshold values. Of the 72 patients with no NAFLD-presence risk factors, only 5 patients (6.9%) had NAFLD. Of the 570 patients with 1 to 4 NAFLD-presence risk factors, 232 patients had NAFLD (40.7%; odds ratio, 9.2). Of the 34 patients with all 5 NAFLD-presence risk factors, 30 patients had NAFLD (88.2%; odds ratio, 100.5).

**Table 1:** Comparison of patients’ characteristics at admission between the two groups

	NAFLD presence	NAFLD absence	|*z*| or chi	*p* value
	*N* = 267	*N* = 409		
Age, years	68 (60, 73)	71 (65, 76)	5.17	< 0.0001
Male sex, *n* (%)	184 (68.9%)	245 (59.9%)	5.71	0.0168
BMI, kg/m^2^	24.8 (22.3, 27)	21.6 (19.5, 24.1)	10.35	< 0.0001
BH, cm	165 (158, 170)	163 (155, 168.3)	3.17	0.0015
BW, kg	67 (58.3, 77)	58 (50, 66)	9.56	< 0.0001
Ischemic stroke, *n* (%)	205 (76.8%)	324 (79.2%)	0.56	0.4536
AST, U/L	24 (20, 31)	22 (19, 28)	3.14	0.0017
ALT, U/L	23 (18, 30)	17 (13, 22)	8.92	< 0.0001
AST/ALT ratio	1.09 (0.86, 1.33)	1.36 (1.10, 1.61)	8.97	< 0.0001
GGT, U/L	32 (21, 59)	24 (16, 42.5)	4.82	< 0.0001
Alb, g/L	42 (40, 44)	40 (37.3, 43)	5.66	< 0.0001
Glucose, mmol/L	7.16 (6.0, 8.88)	6.61 (5.77, 8.22)	2.88	0.0039
HbA1c, % (NGSP)	6 (5.7, 6.7)	5.8 (5.5, 6.2)	4.69	< 0.0001
TC, mmol/L	5.33 (4.58, 6.05)	5.12 (4.43, 5.82)	2.43	0.0150
LDL, mmol/L	3.03 (2.38, 3.71)	2.93 (2.32, 3.47)	1.12	0.2640
HDL, mmol/L	1.38 (1.12, 1.67)	1.53 (1.26, 1.89)	4.66	< 0.0001
TG, mmol/L	1.52 (0.93, 2.64)	1.05 (0.78, 1.57)	6.62	< 0.0001
SBP, mmHg	170.5 (151, 190.3)	162 (141, 182)	3.32	0.0009
DBP, mmHg	97.5 (86, 109.3)	89 (78, 102)	4.96	< 0.0001
MBP, mmHg	120.7 (109.7, 133.2)	113 (102, 128.7)	4.59	< 0.0001
A history of statin use, *n* (%)	60 (22.5%)	96 (23.5%)	0.09	0.7627

All values except for categorical data are represented as median (interquartile range)

*Alb* albumin, *ALT* alanine aminotransferase, *AST* aspartate aminotransferase, *BH* body height, *BMI* body mass index, *BW* body weight, *chi* chi-square value, *DBP* diastolic blood pressure at admission, *GGT* gamma-glutamyl transpeptidase, *HbA1c* glycated hemoglobin, *HDL* high-density lipoprotein cholesterol, *LDL* low-density lipoprotein cholesterol, *MBP* mean blood pressure at admission, *NAFLD* non-alcoholic fatty liver disease, *NGSP* National Glycohemoglobin Standardization Program, *P* probability, *SBP* systolic blood pressure at admission, *TC* total cholesterol, *TG* triglyceride, *|z|* absolute value of the Wilcoxon rank-sum test statistic

**Table 2:** Comparison of serum fatty acids and weight percentages between the two groups

	NAFLD presence	NAFLD absence	|*z*|	*p* value
	*N* = 267	*N* = 409		
*Saturated fatty acids*				
LaA μmol/L	6.99 (3.49, 12.97)	4.99 (2.99, 8.98)	4.13	< 0.0001
MyA μmol/L	94.4 (59.9, 141.1)	68.3 (52.1, 96.8)	6.18	< 0.0001
PA μmol/L	2855 (2321, 3411)	2447 (2127, 2812)	6.63	< 0.0001
StA μmol/L	728.3 (601.7, 835.8)	656.5 (550.5, 766.3)	4.49	< 0.0001
LaA %	0.04 (0.03, 0.08)	0.04 (0.02, 0.06)	2.91	0.0036
MyA %	0.71 (0.53, 0.92)	0.58 (0.47, 0.76)	5.19	< 0.0001
PA %	23.9 (22.9, 25.2)	23.2 (22.2, 24.1)	6.17	< 0.0001
StA %	6.68 (6.20, 7.22)	6.84 (6.22, 7.44)	2.17	0.0298
*n-9 MUFA*				
OlA μmol/L	2381 (1846, 3048)	1993 (1650, 2509)	6.60	< 0.0001
OlA %	22.4 (20.5, 24.6)	20.9 (19.0, 23.0)	6.06	< 0.0001
*n-6 PUFAs*				
LiA μmol/L	2757 (2304, 3156)	2607 (2220, 3080)	2.14	0.0327
DGLA μmol/L	111.2 (86.7, 136.6)	86.4 (68.5, 110.1)	7.76	< 0.0001
AA μmol/L	549.7 (468.7, 648.1)	521.5 (435.7, 617.6)	2.60	0.0092
LiA %	25.3 (22.5, 27.6)	26.9 (24.2, 29.4)	5.90	< 0.0001
DGLA %	1.1 (0.88, 1.31)	0.96 (0.81, 1.16)	4.58	< 0.0001
AA %	5.45 (4.49, 6.32)	5.88 (4.9, 6.74)	3.30	0.0010
*n-3 PUFAs*				
AlA μmol/L	75.4 (54.9, 105.3)	65.7 (49.5, 93.3)	3.24	0.0012
EPA μmol/L	209.2 (138.0, 306.2)	196.3 (133.9, 298.9)	0.61	0.5423
DHA μmol/L	389.7 (315.9, 501.9)	368.1 (286.1, 460.9)	2.66	0.0078
AlA %	0.69 (0.56, 0.85)	0.66 (0.55, 0.86)	0.58	0.5600
EPA %	1.95 (1.33, 3.00)	2.17 (1.44, 3.21)	1.49	0.1369
DHA %	4.30 (3.48, 5.13)	4.49 (3.54, 5.29)	1.43	0.1518
EPA/AA ratio	0.36 (0.23, 0.56)	0.36 (0.26, 0.57)	0.46	0.6468
*n*-6/*n*-3 ratio	4.4 (3.30, 5.56)	4.43 (3.45, 5.56)	0.48	0.6306

All values are represented as median (interquartile range)

*AA* arachidonic acid, *AlA* alpha-linolenic acid, *EPA* eicosapentaenoic acid, *DGLA* dihomo-gamma-linolenic acid, *DHA* docosahexaenoic acid, *LaA* lauric acid, *LiA* linoleic acid, *MyA* myristic acid, *OlA* oleic acid, *P* probability, *PA* palmitic acid, *StA* stearic acid, *NAFLD* non-alcoholic fatty liver disease, *%* weight percentage of total fatty acids, *n-3 PUFA* n-3 polyunsaturated fatty acid, *n-6 PUFA* n-6 polyunsaturated fatty acid, *n-9 MUFA* n-9 monounsaturated fatty acid, |*z*| absolute value of the Wilcoxon rank-sum test statistic

**Table 3:** Multiple logistic regression for NAFLD presence at the onset of stroke using receiver operating characteristic curves (*N* = 676)

	Odds ratio	*p* value	AUC	BIC
		< 0.0001	0.804	803
BMI	1.17 (1.10–1.24)	< 0.0001		
AST/ALT ratio	0.39 (0.23–0.66)	0.0003		
Alb	1.08 (1.03–1.14)	0.0042		
PA%	1.19 (1.03–1.38)	0.0147		
DGLA	1.01 (1.00–1.02)	0.0193		
DHA	1.00 (0.99–1.00)	0.1399		
HbA1c	1.10 (0.98–1.30)	0.1432		
HDL	0.76 (0.45–1.23)	0.3009		
GGT	1.00 (0.99–1.00)	0.3987		
DBP	1.00 (0.99–1.01)	0.4182		
TG	1.09 (0.88–1.37)	0.4547		
MyA%	1.34 (0.59–3.10)	0.4836		
BH	1.01 (0.98–1.03)	0.5109		
StA%	0.96 (0.73–1.27)	0.7937		
AA%	1.02 (0.88–1.18)	0.8224		
Age	0.99 (0.97–1.02)	0.8410		
TC	0.99 (0.80–1.24)	0.9482		

*AA* arachidonic acid, *Alb* albumin, *AUC* area under the curve, *BH* body height, *BIC* Bayesian information criterion, *BMI* body mass index, *DBP* diastolic blood pressure at admission, *DGLA* dihomo-gamma-linolenic acid, *DHA* docosahexaenoic acid, *GGT* gamma-glutamyl transpeptidase, *HDL* high-density lipoprotein cholesterol, *MyA* myristic acid, *P* probability, *PA* palmitic acid, *StA* stearic acid, *TC* total cholesterol, *TG* triglyceride, *%* weight percentage of total fatty acids

**Table 4:** Threshold values for NAFLD presence using receiver operating characteristic curves from logistic regression analysis (*N* = 676)

	Sens (%)	Spec (%)	Odds ratio	*p* value	AUC	BIC
BMI (≥ 23.7 vs. < 23.7) kg/m^2^	65.9	71.4	1.27 (1.21–1.34)	< 0.0001	0.735	808
AST/ALT ratio (≤ 1.25 vs. > 1.25)	70.4	63.1	0.18 (0.11–0.28)	< 0.0001	0.703	848
Alb (≥ 41 vs. < 41) g/L	67.0	52.9	1.11 (1.07–1.16)	< 0.0001	0.628	890
PA% (≥ 24.5 vs. < 24.5)	37.6	84.5	1.39 (1.25–1.55)	< 0.0001	0.641	874
DGLA (≥ 97.1 vs. < 97.1) µmol/L	64.0	67.7	1.02 (1.01–1.02)	< 0.0001	0.676	853

*Alb* albumin, *ALT* alanine aminotransferase, *AST* aspartate aminotransferase, *AUC* area under the curve, *BIC* Bayesian information criterion, *BMI* body mass index, *DGLA* dihomo-gamma-linolenic acid, *P* probability, *PA* palmitic acid, *Sens* sensitivity, *Spec* specificity, % weight percentage of total fatty acids

## Discussion

Our results demonstrate that the US-diagnosed NAFLD frequency in stroke patients aged 40 − 79 years was higher than that in the general Japanese population (39.5% vs. 26.4%, respectively), and the factors associated with NAFLD presence were high BMI, low AST/ALT ratio, high albumin level, high PA%, and high DGLA level. The NAFLD frequency was 88% in acute stroke patients with the five risk factors.

In the present study, the NAFLD frequency was very high in the middle-aged and older acute stroke patients compared to the NAFLD prevalence of 26.4% in the general Japanese population [10]. Blood pressure, glucose level, HbA1c level, total cholesterol, and triglycerides were not independent factors of NAFLD presence in acute stroke patients. Among anthropometric variables, only BMI was an independent factor of NAFLD presence in acute stroke patients. Among liver enzymes, only the AST/ALT ratio was an independent factor. The low AST/ALT ratio was also an independent factor for high BMI. Among variables of serum lipids and fatty acids, neither total cholesterol nor triglycerides, but PA%, a saturated fatty acid, and DGLA, a member of the n-6 PUFA family, were independent NAFLD-presence factors in acute stroke patients. Eicosanoid metabolites of DGLA, such as prostaglandin E1 or thromboxane A1, have anti-inflammatory effects [21]. However, DGLA is metabolized to AA, which is metabolized to prostaglandin E2, thromboxane A2, or leukotriene C4 [22]; these have inflammatory effects and increase the risk of acute myocardial infarction [22]. High serum DGLA level was associated with the onset of acute ischemic stroke and ICH at a young age [17, 18] and was also associated with obesity, body fat accumulation, high ALT level, and insulin resistance in patients with type 2 diabetes [23]. High serum DGLA level was associated with not only acute stroke but also NAFLD presence. In a previous study, low fish intake was associated with high DGLA% [24]. The dietary effect of DGLA may be more profound than that of gamma-linolenic acid, which is enzymatically elongated to DGLA [25]. Therefore, diets that contain large amounts of DGLA may increase serum DGLA levels. For example, Japanese beef cattle or crossbred (Holstein × Japanese black) beef steer contains more DGLA than imported beef or lamb [26]. However, it has not been investigated whether serum DGLA levels increase in proportion to the consumption of these proteins and whether high DGLA levels cause obesity and NAFLD.

In Japan, the National Health and Nutrition Survey reported that mean BMI slightly increased in men and women from 1995 to 2016 and the total energy intake and energy intake from proteins decreased, whereas energy intake from fats increased from 1995 to 2016 [27]. Obesity is a stubbornly obvious target for stroke prevention [28]. Therefore, a study was conducted to investigate whether the Japanese diet, characterized by high seafood and plant food composition, affects fatty acid intake and serum fatty acid% [29]. It was found that the Japanese diet, which comprises fish, soybeans, soy products, seaweed, mushroom, konjac, and unrefined cereals, with low amounts of animal fat, meat, poultry with fat, and sweets, including deserts and snacks, was effective in increasing serum n-3 PUFA% and decreasing serum *n*-6 PUFA% [29]. Serum DGLA% had a strong positive correlation with serum DGLA level (Supplemental Table 8). Therefore, the Japanese diet may decrease serum DGLA level as a member of the *n*-6 PUFA family. The Japanese diet successfully decreased BW, BMI, and systolic and diastolic blood pressure [30]. If serum DGLA level is decreased, the incidence of NAFLD and obesity is reduced, and the incidence of an acute stroke may be prevented.

### Limitations

Our study had several limitations. First, a small number of patients were included, and the study was based on a retrospective cross-sectional design without control. We compared acute stroke patients with NAFLD to those without NAFLD but did not compare them with a control population. Furthermore, a cross-sectional study cannot exclude reverse causality. Second, although ultrasonography has a sensitivity of 100% in detecting > 33% of fat, ultrasonography does not always find ≤ 33% fat and cannot distinguish steatohepatitis from simple steatosis [19, 20, 31]. Third, the patients’ self-reported alcohol intake might have been biased. Fourth, because most of the patients were Japanese and there might be racial differences in the NAFLD-presence factors and the threshold values of the NAFLD-presence factors, the generalizability of the study outcomes to non-Japanese populations is uncertain. Therefore, a prospective study, including a food frequency questionnaire for detailed history of health supplement use, accurate anthropometric measurements, and biomarkers such as serum AST, ALT, albumin, fatty acid levels, and fatty acid%, is required to determine the effects of the NAFLD-presence factors on stroke onset or prevention.

## Conclusions

In acute stroke patients aged 40 − 79 years, the NAFLD frequency of 39.5% at acute stroke admission was very high. The factors associated with NAFLD-presence were high BMI, low AST/ALT ratio, high albumin level, high PA% level, and high DGLA level. A further study is warranted to determine the effects of the NAFLD-presence factors on stroke onset or prevention.

## Supplementary Information

Below is the link to the electronic supplementary material.

Supplementary file1 (PDF 180 KB)

## Data Availability

The datasets generated during and/or analyzed during the current study are available from the corresponding author on reasonable request.
