# Supplementary material for: Non-alcoholic fatty liver disease frequency and associated factors at admission of acute stroke
Source: Hepatol Int. 2021 Sep 15;16(1):81–8. doi: 10.1007/s12072-021-10253-z (PMC8844138; doi:10.1007/s12072-021-10253-z)
Supplement: Supplementary file 1 — Supplementary file1 (PDF 180 KB) [file 12072_2021_10253_MOESM1_ESM.pdf]

SUPPLEMENTAL MATERIAL

Supplemental Table 1 Patients' characteristics (N=676)

Variables	Values
NAFLD, n (%)	267 (39.5%)
Male sex, n (%)	429 (63.5%)
Age, years	70 (63, 75)
BMI, kg/m^2	22.9 (20.5, 25.5)
BH, cm	164 (157, 169)
BW, kg	61 (53, 70)
Alb, g/L	41 (38, 43)
AST, U/L	23 (19, 29)
ALT, U/L	19 (14,26)
AST/ALT ratio	1.25 (1.0, 1.53)
GGT, U/L	26.5 (18, 48)
Glucose, mmol/L	6.77 (5.83, 8.44)
HbA1c, % (NGSP)	5.9 (5.6, 6.4)
TC, mmol/L	5.18 (4.47, 5.92)
LDL, mmol/L	2.95 (2.33, 3.55)
HDL, mmol/L	1.47 (1.20, 1.81)
TG, mmol/L	1.16 (0.82, 1.90)
SBP, mmHg	165 (145, 187)
DBP, mmHg	93 (81, 105)
MBP, mmHg	116.7 (103.7, 130.7)

All values except for categorical data are represented as median (interquartile range). Abbreviations: ALT, alanine aminotransferase; AST, aspartate aminotransferase; BH, body height; Bold values denote significant correlation; BW, body weight; DBP, diastolic blood pressure at admission; DHA, docosahexaenoic acid; DGLA, dihomo-gamma-linolenic acid; GGT, gamma-glutamyl transpeptidase; HbA1c, glycated hemoglobin; HDL, high-density lipoprotein cholesterol; LDL, low-density lipoprotein cholesterol; MBP, mean blood pressure at admission; NAFLD, non-alcoholic fatty liver disease; NGSP, National Glycohemoglobin Standardization Program; SBP, systolic blood pressure at admission; $r_{s,}$ Spearman correlation coefficient; TC, total cholesterol; TG, triglyceride.

Supplemental Table 2 Serum fatty acids and weight percentages of total fatty acids

Fatty acids	Values
**Saturated fatty acids**	
LaA, µmol/L	5.49 (2.99, 10.48)
MyA, µmol/L	77.1 (55.6, 112.6)
PA, µmol/L	2583 (2186, 3066)
StA, µmol/L	682 (567.4, 798.2)
LaA, %	0.04 (0.02, 0.07)
MyA, %	0.62 (0.49, 0.83)
PA, %	23.5 (22.5, 24.5)
StA, %	6.78 (6.20, 7.34)
**n-9 MUFA**	
OlA, µmol/L	2117 (1734, 2721)
OlA, %	21.6 (19.5, 23.7)
**n-6 PUFAs**	
LiA, µmol/L	2656 (2262, 3112)
DGLA, µmol/L	94.2 (75.0, 121.3)
AA, µmol/L	529.6 (447.8, 626.2)
LiA, %	26.2 (23.5, 28.5)
DGLA, %	1.03 (0.84, 1.21)
AA, %	5.67 (4.77, 6.56)
**n-3 PUFAs**	
AlA, µmol/L	68.9 (52.1, 97.6)
EPA, µmol/L	202.4 (134.5, 303.4)
DHA, µmol/L	377.9 (295.9, 470.8)
AlA, %	0.68 (0.56, 0.85)
EPA, %	2.1 (1.39, 3.11)
DHA, %	4.44 (3.51, 5.26)
EPA/AA ratio	0.36 (0.25, 0.56)
n-6/n-3 ratio	4.41 (3.37, 5.56)

All values are represented as median (interquartile range). AA, arachidonic acid; AlA, alpha-linolenic acid; DGLA, Dihomo-gamma-linolenic acid; DHA, docosahexaenoic acid; EPA, eicosapentaenoic acid; LaA, lauric acid; LiA, linoleic acid; MyA, myristic acid; n-3 PUFA, n-3 polyunsaturated fatty acid; n-6 PUFA, n-6 polyunsaturated fatty acid; n-9 MUFA, n-9 monounsaturated fatty acid; OlA, oleic acid; PA, palmitic acid; StA, stearic acid; %: weight percentage of total fatty acids.

Supplemental Table 3 Spearman's rank correlation coefficients between male sex and anthropometric variables

r_s	BH	BW	BMI	Male sex
BH		**0.62**	0.16	**0.72**
BW	**0.62**		**0.85**	**0.51**
BMI	0.16	**0.85**		0.19
Male sex	**0.72**	**0.51**	0.19	

Boldface indicates strong correlation.

BH, body height; BMI, body mass index; BW, body weight;

Bold values denote strong correlation; $r_{s,}$ Spearman's rank correlation coefficient;

Supplemental Table 4 Spearman's rank correlation coefficients between age, serum albumin, glucose, and lipid levels

r_s	Age	Alb	Glucose	HbA1c	TC	LDL	HDL	TG
Age		-0.23	0.07	0.11	-0.19	-0.09	-0.05	-0.15
Alb	-0.23		0.00	-0.03	0.31	0.23	0.16	0.08
Glucose	0.07	0.00		**0.53**	0.03	0.04	-0.12	0.10
HbA1c	0.11	-0.03	**0.53**		0.06	0.09	-0.14	0.14
TC	-0.19	0.31	0.03	0.06		**0.83**	0.41	0.22
LDL	-0.09	0.23	0.04	0.09	**0.83**		0.16	0.00
HDL	-0.05	0.16	-0.12	-0.14	0.41	0.16		-0.35
TG	-0.15	0.08	0.10	0.14	0.22	0.00	-0.35	

Boldface indicates strong correlation. Alb, albumin; HbA1c, glycated hemoglobin; HDL, high-density lipoprotein cholesterol; LDL, low-density lipoprotein cholesterol; r_s, Spearman's rank correlation coefficient; TC, total cholesterol; TG, triglycerides

Supplemental Table 5 Spearman's rank correlation coefficients between variables of transaminase

r_s	AST	ALT	AST/ALT ratio	GGT
AST		**0.70**	0.02	0.42
ALT	**0.70**		**-0.63**	0.48
AST/ALT ratio	0.02	**-0.63**		-0.23
GGT	0.42	0.48	-0.23	

Boldface indicates strong correlation.

ALT, alanine aminotransferase; AST, aspartate aminotransferase;

GGT, gamma-glutamyl transpeptidase;

r_s, Spearman's rank correlation coefficient

Supplemental Table 6 Spearman's rank correlation coefficients between variables of blood pressure

r_s	SBP	DBP	MBP
SBP		**0.60**	**0.87**
DBP	**0.60**		**0.91**
MBP	**0.87**	**0.91**	

Boldface indicates strong correlation.

DBP, diastolic blood pressure at admission;

MBP, mean blood pressure at admission;

SBP, systolic blood pressure at admission;

$r_{s,}$ Spearman's rank correlation coefficient

Supplemental Table 7 Spearman's rank correlation coefficients between serum fatty acids

r_s	LaA	MyA	PA	StA	OlA	LiA	DGLA	AA	AlA	EPA	DHA	EPA/ AA	n-6/n-3
LaA		**0.84**	**0.55**	**0.61**	**0.53**	0.49	0.44	0.08	**0.56**	-0.05	0.06	-0.08	0.19
MyA	**0.84**		**0.77**	**0.76**	**0.73**	**0.59**	**0.58**	0.21	**0.70**	0.09	0.28	-0.00	0.07
PA	**0.55**	**0.77**		**0.81**	**0.91**	**0.71**	**0.60**	0.47	**0.66**	0.12	0.45	-0.08	0.06
StA	**0.61**	**0.76**	**0.81**		**0.74**	**0.73**	**0.61**	0.46	**0.65**	0.16	0.42	-0.03	0.06
OlA	**0.53**	**0.73**	**0.91**	**0.74**		**0.64**	**0.59**	0.38	**0.66**	-0.00	0.32	-0.16	0.15
LiA	0.49	**0.59**	**0.71**	**0.73**	**0.64**		0.49	0.32	**0.73**	-0.06	0.23	-0.19	0.37
DGLA	0.44	**0.58**	**0.60**	**0.61**	**0.59**	0.49		0.41	0.40	-0.22	0.06	-0.38	0.36
AA	0.08	0.21	0.47	0.46	0.38	0.32	0.41		0.16	0.16	0.35	-0.24	0.02
AlA	**0.56**	**0.70**	**0.66**	**0.65**	**0.66**	**0.73**	0.40	0.16		0.09	0.31	0.02	0.08
EPA	-0.05	0.09	0.12	0.16	-0.00	-0.06	-0.22	0.16	0.09		**0.68**	**0.90**	**-0.87**
DHA	0.06	0.28	0.45	0.42	0.32	0.23	0.06	0.35	0.31	**0.68**		**0.52**	**-0.68**
EPA/AA	-0.08	-0.00	-0.08	-0.03	-0.16	-0.19	-0.38	-0.24	0.02	**0.90**	**0.52**		**-0.87**
n-6/n-3	0.19	0.07	0.06	0.06	0.15	0.37	0.36	0.02	0.08	**-0.87**	**-0.68**	**-0.87**	

Boldface indicates strong correlation.

AA, arachidonic acid; AlA, alpha-linolenic acid; DHA, docosahexaenoic acid; DGLA, dihomo-gamma-linolenic acid; EPA, eicosapentaenoic acid; LaA, lauric acid; LiA, linoleic acid; MyA, myristic acid; OlA, oleic acid; PA, palmitic acid; StA, stearic acid; Bold values, significant correlation; r_s, Spearman's rank correlation coefficient; n-3, n-3 polyunsaturated fatty acids; n-6, n-6 polyunsaturated fatty acids

Supplemental Table 8 Spearman's rank correlation coefficients between serum fatty acid weight percentages

r_s	LaA %	MyA %	PA %	StA %	OlA %	LiA %	DGLA %	AA %	AlA %	EPA %	DHA %
LaA %		**0.80**	0.18	0.26	0.23	-0.00	0.12	-0.40	0.37	-0.22	-0.33
MyA %	**0.80**		0.26	0.19	0.30	0.17	0.17	0.47	0.42	-0.15	-0.23
PA %	0.18	0.26		-0.26	-0.30	-0.51	0.08	-0.19	-0.15	-0.17	-0.12
StA %	0.26	0.19	-0.26		-0.30	0.07	0.19	0.06	-0.00	0.07	0.02
OlA %	0.23	0.30	-0.30	-0.30		-0.43	0.05	-0.40	0.16	-0.44	-0.42
LiA %	-0.00	0.17	**-0.51**	0.07	-0.43		0.02	-0.10	0.23	-0.27	-0.24
DGLA %	0.12	0.17	0.08	0.19	0.05	0.02		0.14	-0.13	-0.38	-0.32
AA %	-0.40	0.47	-0.19	0.06	-0.40	-0.10	0.14		-0.49	0.18	0.23
AlA %	0.37	0.42	-0.15	-0.00	0.16	0.23	-0.13	-0.49		-0.11	-0.14
EPA %	-0.22	-0.22	-0.17	0.07	-0.44	-0.27	-0.38	0.18	-0.11		**0.73**
DHA %	-0.33	-0.33	-0.12	0.02	-0.42	-0.24	-0.32	0.23	-0.14	**0.73**	

Boldface indicates strong correlation.

AA, arachidonic acid; AlA, alpha-linolenic acid; DHA, docosahexaenoic acid; DGLA, dihomo-gamma-linolenic acid; EPA, eicosapentaenoic acid; LaA, lauric acid; LiA, linoleic acid; MyA, myristic acid; OlA, oleic acid; PA, palmitic acid; StA, stearic acid; Bold values, significant correlation; r_{s}, Spearman's rank correlation coefficient, %, weight percentage of total fatty acids.

Supplemental Table 9 Spearman's rank correlation coefficients between anthropometric variables, hepatic variables, and diastolic blood pressure

r_s	BMI	BH	AST/ALT ratio	GGT	DBP
BMI		0.16	-0.38	0.17	0.15
BH	0.16		-0.14	0.21	0.09
AST/ALT ratio	-0.38	-0.38		-0.23	-0.05
GGT	0.17	0.17	-0.23		0.17
DBP	0.15	0.15	-0.05	0.17	

ALT, alanine aminotransferase; AST, aspartate aminotransferase; BH, body height;
DBP, diastolic blood pressure; GGT, gamma-glutamyl transpeptidase;
r_s, Spearman's rank correlation coefficient.

Supplemental Table 10 Spearman's rank correlation coefficients between selected fatty acids and fatty acid percentages

r_s	MyA%	PA%	StA%	OlA%	DGLA%	AA%
DGLA	0.43	0.21	0.08	0.37	**0.74**	-0.16
AA	-0.02	0.03	-0.03	0.07	0.11	**0.53**
DHA	0.10	0.06	-0.07	-0.04	-0.31	-0.06

Boldface indicates strong correlation.

AA, arachidonic acid; DHA, docosahexaenoic acid; DGLA, dihomo-gamma-linolenic acid;

MyA, myristic acid; OlA, oleic acid; PA, palmitic acid; StA, stearic acid;

r_s, Spearman's rank correlation coefficient; %, weight percentage of total fatty acids

Supplemental Table 11 Spearman's rank correlation coefficients between age, selected serum variables, and fatty acids

r_s	DGLA	DHA	MyA%	PA%	StA%	OlA%	AA%
Age	-0.32	0.09	-0.07	-0.06	-0.06	-0.16	0.04
Alb	0.20	0.11	0.08	-0.05	0.01	-0.07	0.04
HbA1c	0.04	0.06	0.01	0.16	-0.17	0.07	-0.07
TC	0.38	0.37	0.08	-0.08	0.06	-0.16	-0.01
HDL	-0.01	0.14	-0.09	-0.16	0.35	-0.45	0.24
TG	0.48	0.27	-0.09	0.31	-0.20	**0.59**	-0.37

AA, arachidonic acid; Alb, albumin; DHA, docosahexaenoic acid; DGLA, dihomo-gamma-linolenic acid; HbA1c, glycated hemoglobin; HDL-C, high-density lipoprotein cholesterol; MyA, myristic acid; OlA, oleic acid; PA, palmitic acid; StA, stearic acid; r_s, Spearman's rank correlation coefficient; TC, total cholesterol; TG, triglyceride; %, weight percentage of total fatty acids.

Supplemental Table 12 Spearman's rank correlation coefficients between anthropometric variables, age, selected serum variables, and fatty acids

r_s	Age	Alb	HbA1c	TC	HDL	TG	DGLA	DHA	MyA%	PA%	StA%	AA%
BMI	-0.19	0.12	0.23	0.01	-0.27	0.27	0.28	0.04	0.11	0.16	-0.17	-0.10
BH	-0.18	0.00	0.07	-0.18	-0.19	0.09	0.00	-0.11	0.02	0.20	-0.08	-0.03
AST/ALT ratio	0.27	-0.21	-0.20	-0.05	0.16	-0.16	-0.20	-0.01	-0.13	-0.09	-0.02	0.03
GGT	-0.13	-0.04	0.08	-0.03	-0.07	0.27	0.18	0.08	0.15	0.32	0.01	-0.12
DBP	-0.29	0.27	-0.02	0.23	0.09	0.18	0.26	0.05	0.14	0.08	0.03	-0.12

AA, arachidonic acid; Alb, albumin; ALT, alanine aminotransferase; AST, aspartate aminotransferase; BH, body height; DBP, diastolic blood pressure; DHA, docosahexaenoic acid; DGLA, dihomo-gamma-linolenic acid; GGT, gamma-glutamyl transpeptidase; HbA1c, glycated hemoglobin; HDL, high-density lipoprotein cholesterol; MyA, myristic acid; PA, palmitic acid; StA, stearic acid; r_s, Spearman's rank correlation coefficient; TC, total cholesterol; TG, triglyceride; %, weight percentage of total fatty acids.

Supplemental Table 13 Variance inflation factor of variables for multiple logistic regression analysis

	VIF
BMI	1.31
AST/ALT ratio	1.25
Alb	1.38
PA%	1.46
DGLA	1.97
DHA	1.33
HbA1c	1.07
HDL	1.71
GGT	1.14
DBP	1.26
TG	1.90
MyA%	1.85
BH	1.18
StA%	1.40
AA%	1.37
Age	1.35
TC	1.91

AA, arachidonic acid; Alb, albumin; AUC, area under the curve; BH, body height; BIC, Bayesian information criterion; BMI, body mass index; DBP, diastolic blood pressure at admission; DGLA, dihomo-gamma-linolenic acid; DHA, docosahexaenoic acid; GGT, gamma-glutamyl transpeptidase; HbA1c, glycated hemoglobin; HDL, high-density lipoprotein cholesterol; MyA, myristic acid; *P*, probability; PA, palmitic acid; StA, stearic acid; TC, total cholesterol; TG, triglyceride; VIF, variance inflation factor; %, weight percentage of total fatty acids.
